# Spatial Information-Theoretic Optimal LPI Radar Waveform Design

**DOI:** 10.3390/e24111515

**Published:** 2022-10-24

**Authors:** Jun Chen, Jie Wang, Yidong Zhang, Fei Wang, Jianjiang Zhou

**Affiliations:** 1 School of Electronic and Information Engineering, Nanjing University of Information Science and Technology, Nanjing 210044, China; 2 Key Laboratory of Radar Imaging and Microwave Photonics, Ministry of Education, Nanjing University of Aeronautics and Astronautics, Nanjing 210016, China

**Keywords:** LPI, radar waveform, passive interception systems, Kullback–Leibler divergence, joint entropy

## Abstract

In this paper, the design of low probability of intercept (LPI) radar waveforms considers not only the performance of passive interception systems (PISs), but also radar detection and resolution performance. Waveform design is an important considerations for the LPI ability of radar. Since information theory has a powerful performance-bound description ability from the perspective of information flow, LPI waveforms are designed in this paper within the constraints of the detection performance metrics of radar and PISs, both of which are measured by the Kullback–Leibler divergence, and the resolution performance metric, which is measured by joint entropy. The designed optimization model of LPI waveforms can be solved using the sequential quadratic programming (SQP) method. Simulation results verify that the designed LPI waveforms not only have satisfactory target-detecting and resolution performance, but also have a superior low interception performance against PISs.

## 1. Introduction

Low probability of intercept (LPI) radar waveforms have been developed to combat passive interception systems (PISs) for several decades [1,2,3]. Common LPI waveforms include FM/PM signals, FSK/PSK signals, etc. [2,4,5,6,7], which utilize wideband modulations to spread the energy in a frequency. LPI radar waveform design is a primary means of affecting the interception performance of PISs, which is actually a compromise between radar performance (which contains detection and resolution performance) and the interception performance of the PIS. In this paper, the dispersion of waveform energy in a frequency can be implemented through the compromise between the optimal detection performance, resolution performance, and LPI performance of radar by adjusting their frequency amplitudes.

In frequency amplitude adjustment modeling, it is crucial to establish the performance metrics of both the radar and the PIS. For radar detection performance, besides metrics such as output signal-to-noise ratio (SNR), relative entropy, and mean square error, the method of maximizing mutual information has been widely used in optimal radar waveform design (see [8,9,10,11] and references therein). Zhu et al. [12] presented the Kullback–Leibler divergence (KLD) as more appropriate than mutual information to describe optimal radar detection performance. The KLD is defined as

(1) $$D\left(x;n_{1}\right)=E_{y}\left[D\left(x;n_{1}|y\right)\right]-I\left(x;y\right),$$

where $x=y+n_{1}$ is the received radar signal, y is the target response, $n_{1}$ is the background radar noise, $E\left(\cdot \right)$ denotes capture expectation, $I\left(\cdot \right)$ denotes mutual information, and $D\left(\cdot \right)$ denotes the KLD. For radar resolution performance, the most classic and common metric is the ambiguity function [13]. In this paper, we will design a simpler resolution metric for radar by using joint entropy. For PISs, the common interception performance metric includes the peak-to-average power ratio, time–bandwidth product, and so on (see [2,7,14] and references therein). Here, we regard the KLD, denoted by $D\left(z;n_{2}\right)$, between the intercept signal z and background noise $n_{2}$ of a PIS as the effective interception performance metric of a PIS for LPI radar waveform design. Thus, by maximizing the detection and resolution performance of radar and minimizing the interception performance of the PIS, an optimization problem of frequency amplitudes can be established and solved with the constraint of a fixed transmission power.

## 2. LPI Radar Waveform Design Method

We presume the real radar signal $s\left(t\right)$ is emitted by the transmitting antenna with gain $G_{t}$ in the target direction. It can be scattered and intercepted by the target, which is equipped with a PIS. We denote $x\left(t\right)=y\left(t\right)+n_{1}\left(t\right)$ as the signal received by the receiving antenna of the radar with gain $G_{r}$, where $y\left(t\right)=\alpha s\left(t\right)*h\left(t\right)$ is the target response, $h\left(t\right)$ is the target impulse response, $\alpha =\sqrt{\frac{G_{t}G_{r}\lambda^{2}L_{1}}{{\left(4\pi \right)}^{3}R^{4}}}$ is the energy attenuation coefficient, λ is the wavelength, R is the distance to the target, $L_{1}$ is the total radar path loss, and the symbol “∗“ denotes convolution. Meanwhile, the signal intercepted by the receiving antenna of the PIS with gain $G_{i}$ can be denoted as $z\left(t\right)=\beta s\left(t\right)+n_{2}\left(t\right)$, where $\beta =\sqrt{\frac{G_{t}G_{i}\lambda^{2}L_{2}}{{\left(4\pi \right)}^{2}R^{2}}}$, $L_{2}$ is the path loss between radar and target. We also assume that $h\left(t\right)$, $n_{1}\left(t\right)$, and $n_{2}\left(t\right)$ are zero-mean Gaussian random processes.

For the convenience of analysis, we split the frequency interval W of the radar waveform into a large number of sufficiently small and disjointed frequency intervals $F_{k}=\left[f_{k},f_{k}+\delta f\right]$, so that for all $f\in F_{k}$, we have $S\left(f\right)\approx S\left(f_{k}\right)$, where $S\left(f\right)$ is the frequency domain waveform of $s\left(t\right)$. We denote $z_{k}$ and $n_{2,k}$ as components of z and $n_{2}$, with frequencies in $F_{k}$, where z is the received signal vector of the PIS whose elements are the samplings of $z\left(t\right)$, and $n_{2}$ is the corresponding background noise vector of the PIS. Based on sampling theory, we suppose the sampling frequency is 2δf, and therefore the sample size is 2δfT, where T denotes the duration of signals.

According to the expressions of the KLD between two Gaussian probability density functions (PDFs) and the entropy of a Gaussian random variable, the terms in Equation (1) can be calculated as

(2) $$E_{y}\left[D\left(x;n_{1}|y\right)\right]=T\int_{W}\frac{2\alpha^{2}|S\left(f\right){|}^{2}\sigma_{H}^{2}\left(f\right)}{TP_{N_{1}}\left(f\right)}df,$$

(3) $$I\left(x;y\right)=T\int_{W}\ln\left[1+\frac{2\alpha^{2}|S\left(f\right){|}^{2}\sigma_{H}^{2}\left(f\right)}{TP_{N_{1}}\left(f\right)}\right]df,$$

where $\sigma_{H}^{2}\left(f\right)$ is the variance of $H\left(f\right)$, which is the Fourier transform of $h\left(t\right)$, and $P_{N_{1}}\left(f\right)$ is the one-sided power spectral density (PSD) of $n_{1}\left(t\right)$.

Thus, the KLD between x and $n_{1}$ can be written as

(4) $$D\left(x;n_{1}\right)=T\int_{W}\left\{\frac{2\alpha^{2}|S\left(f\right){|}^{2}\sigma_{H}^{2}\left(f\right)}{TP_{N_{1}}\left(f\right)}-\ln\left[1+\frac{2\alpha^{2}|S\left(f\right){|}^{2}\sigma_{H}^{2}\left(f\right)}{TP_{N_{1}}\left(f\right)}\right]\right\}df.$$

In the design of LPI radar waveforms, the resolution performance should also be considered, which is another quite important performance measure for radar. In this paper, we use autocorrelation to describe radar resolution performance, which is a more concise way of representing the ambiguity function. For time, the worse the autocorrelation is, the better the range resolution is. Correspondingly, for frequency, the worse the autocorrelation is, the better the velocity resolution is. Next, we will utilize joint entropy to describe the autocorrelation.

Suppose the samplings of the transmitted waveform $s={\left[s\left(1\right),s\left(2\right),\cdots ,s\left(L\right)\right]}^{T}$ come from the normal distribution with mean 0 and variance $\sigma_{l}^{2}$. Then, the entropy of sample $s\left(l\right)$ can be computed as

(5) $$\mathrm{Entropy}\left[s\left(l\right)\right]=\frac{1}{2}\left[\log2\pi +1\right]+\frac{1}{2}\log\sigma_{l}^{2}.$$

Since the joint probability density function of these samples is $p\left(s\right)={\left(2\pi \right)}^{L}|R{|}^{-\frac{1}{2}}\exp\left(-\frac{1}{2}s^{T}|R{|}^{-1}s\right)$, the joint entropy of these samples can be calculated as

(6) $$\mathrm{Entropy}\left[s\right]=\frac{L}{2}\left[\log2\pi +1\right]+\frac{1}{2}\log|R|,$$

where R is the sample covariance matrix of transmitted waveform s, which can be estimated as $\hat{R}={\left(r_{i,j}\right)}_{i=1,j=1}^{L,L}$, $r_{i,j}=\frac{1}{L}\sum_{l=1}^{L-|i-j|}s\left(l\right)s\left(l+|i-j|\right)$, and R is a Toeplitz and symmetric matrix.

If the designed waveform has a perfect resolution performance, then the samples are independent. It means that the joint entropy is equal to the sum of the entropy of each sample, that is: $\mathrm{Entropy}\left[s\right]=\sum_{l=1}^{L}\mathrm{Entropy}\left[s\left(l\right)\right]$. In fact, the designed waveform cannot have a perfect resolution performance. The joint entropy and the sum of the entropy of each sample have a relationship as follows: $\mathrm{Entropy}\left[s\right]\leq \sum_{l=1}^{L}\mathrm{Entropy}\left[s\left(l\right)\right]$. Therefore, we use the difference between the joint entropy and the sum of the entropy of each sample as the metric of resolution performance, which can be expressed as

(7) $$\Delta E_{s}=\sum_{l=1}^{L}\mathrm{Entropy}\left[s\left(l\right)\right]-\mathrm{Entropy}\left[s\right]=\frac{1}{2}\sum_{l=1}^{L}\log\sigma_{l}^{2}-\frac{1}{2}\log|R|,$$

where $\Delta E_{s}\geq 0$.

From Equation (7), we find that the smaller the value of the metric, the better the resolution performance of the transmitted waveform, and when the transmitted waveform is white Gaussian noise, the value of the metric $\Delta E_{s}$ is equal to zero, which means the white Gaussian noise has a perfect resolution performance. In the design of LPI waveforms, we need to minimize $\Delta E_{s}$. For solving it conveniently, since R is a Toeplitz and symmetric matrix, we can simplify the metric $\Delta E_{s}$ to a convex function, which can be expressed as

(8) $$\overset{⏜}{\Delta E_{s}}=\frac{\sum_{j=2}^{c}r_{1,j}}{(c-1)P_{S}}=\frac{\sum_{j=2}^{c}\sum_{l=1}^{L-|1-j|}s\left(l\right)s\left(l+|1-j|\right)}{(c-1)LP_{S}},$$

where c is the constraint number of the time delay or Doppler shift, which can be set according to the practical application, since we do not need to constrain the autocorrelation for each time delay and Doppler shift, and $P_{S}$ is the average power spectral density of the transmitted waveform, which is used for the purpose of normalization, that is, the upper bound of $\overset{⏜}{\Delta E_{s}}$ is equal to one.

For frequency, resolution performance has the same computational procedure. We only need to substitute $s\left(l\right)$ with $S\left(f_{l}\right)$, and we denote the metric of resolution performance for frequency as $\overset{⏜}{\Delta E_{S}}$.

It is a common view that white noise is the best LPI waveform. The closer the distance between intercept signal z and background noise $n_{2}$, the more difficult it is to detect and recognize the intercept signal. The KLD has been confirmed to be a powerful and accurate tool to measure the information of multivariate data, with lesser complexity and superior performance among the existing distance measures, such as $L^{1}$, Bhattacharyya distance, Hellinger distance, f− divergence, etc. [15,16,17]. Therefore, here we use KLD as the PDF distance measure, which is denoted as $D\left(z;n_{2}\right)$.

The KLD between $z_{k}^{m}$ and $n_{2,k}^{m}$, which are samples of $z_{k}$ and $n_{2,k}$ in each frequency $f_{m}\in F_{k}$ can be written as $D\left(z_{k}^{m};n_{2,k}^{m}\right)=\frac{\beta^{2}|S\left(f_{k}\right){|}^{2}}{P_{N_{2}}\left(f_{k}\right)}$, where $P_{N_{2}}\left(f\right)$ is the one-sided power spectral density (PSD) of $n_{2}\left(t\right)$, that is a function of SNR. The lower SNR a waveform possesses in each frequency, the harder it is for a PIS to intercept it. This agrees with our common knowledge and experience. In order to solve the following optimization problem smoothly, we take the natural exponential function of $D\left(z_{k}^{m};n_{2,k}^{m}\right)$, which can maintain the monotonicity near $|S\left(f_{k}\right){|}^{2}$. It can be denoted as $\tilde{D}\left(z_{k}^{m};n_{2,k}^{m}\right)=\exp\left\{\frac{\beta^{2}|S\left(f_{k}\right){|}^{2}}{P_{N_{2}}\left(f_{k}\right)}\right\}$. Thus, the modified KLD between component $z_{k}$ and $n_{2,k}$ is

(9) $$D\left(z_{k};n_{2,k}\right)=2\delta f\;T\tilde{D}\left(z_{k}^{m};n_{2,k}^{m}\right)=2\delta fT\exp\left\{\frac{\beta^{2}|S\left(f_{k}\right){|}^{2}}{P_{N_{2}}\left(f_{k}\right)}\right\}.$$

When δf→0, the modified KLD between z and $n_{2}$ can be obtained as

(10) $$D\left(z;n_{2}\right)=2T\int_{W}\exp\left\{\frac{\beta^{2}|S\left(f\right){|}^{2}}{P_{N_{2}}\left(f\right)}\right\}df.$$

Since the KLDs $D\left(x;n_{1}\right)$ and $D\left(z;n_{2}\right)$ can be used to measure the detection performance of radar and a PIS respectively, and $\overset{⏜}{\Delta E_{s}}$ and $\overset{⏜}{\Delta E_{S}}$ can be used to measure the resolution performance of radar, we can design an LPI radar waveform which not only has superior target detecting and resolution performance, but also has superior LPI performance against PIS interception based on these four metrics. The optimization problem of LPI radar waveform design can be straightforwardly described as $s^{⋆}=arg\left\{\max_{s}D\left(x;n_{1}\right),\min_{s}\overset{⏜}{\Delta E_{s}},\min_{s}\overset{⏜}{\Delta E_{S}},\min_{s}D\left(z;n_{2}\right)\right\}$, under the constraint that the average transmitted power is fixed, denoted by $\int_{W}|S\left(f\right){|}^{2}df=P_{s}$.

LPI radar waveform design is a trade-off between the performance of radar and of PISs, which is to maximize the detection and resolution performance of radar and minimize the interception performance of PISs. In fact, the primary task of the emitted waveform is to accomplish target detection and resolution. The designed radar waveform should be considered for its LPI capability under the condition of meeting radar performance. Therefore, we minimize the KLD $D\left(z;n_{2}\right)$ of the PIS in the situation that the KLD $D\left(x;n_{1}\right)$, $\overset{⏜}{\Delta E_{s}}$, and $\overset{⏜}{\Delta E_{S}}$ of radar make some concessions, which can be expressed as

(11) $$\begin{array}{c} s^{⋆}=arg\left\{\underset{s}{\min}D\left(z\;\;;n_{2}\right)\right\}\\ s.t.\;D\left(x;n_{1}\right)\geq \gamma ,\;\;\;\;\overset{⏜}{\Delta E_{s}}\leq \nu_{1},\;\;\;\;\overset{⏜}{\Delta E_{S}}\leq \nu_{2},\;\;\;\;\int_{W}|S\left(f\right){|}^{2}df=P_{s}, \end{array}$$

where γ is the value of $D\left(x;n_{1}\right)$ required to meet radar detection performance, and $\nu_{1}$ and $\nu_{2}$ are the values of $\overset{⏜}{\Delta E_{s}}$ and $\overset{⏜}{\Delta E_{S}}$ needed to meet the radar resolution performance for range and velocity, respectively. All of them can be set to various values in different competing scenarios.

In order to achieve the optimal solution of Equation (11), the discrete form of the optimization problem first needs to be obtained, which can be written as

(12) $$\;\;s^{⋆}=arg\left\{\underset{s}{\min}2Tb\sum_{l=1}^{L}e^{\frac{\beta^{2}|S\left(f_{l}\right){|}^{2}}{P_{N_{2}}\left(f_{l}\right)}}\right\},\;s.t.\{\begin{array}{c} Tb\sum_{l=1}^{L}\left\{\frac{2\alpha^{2}|S\left(f_{l}\right){|}^{2}\sigma_{H}^{2}\left(f_{l}\right)}{TP_{N_{1}}\left(f_{l}\right)}-\ln\left[1+\frac{2\alpha^{2}|S\left(f_{l}\right){|}^{2}\sigma_{H}^{2}\left(f_{l}\right)}{TP_{N_{1}}\left(f_{l}\right)}\right]\right\}\geq \gamma \\ \frac{\sum_{j=2}^{c}\sum_{l=1}^{L-|1-j|}s\left(l\right)s\left(l+|1-j|\right)}{(c-1)LP_{S}}\leq \nu_{1},\;\;\frac{\sum_{j=2}^{c}\sum_{l=1}^{L-|1-j|}S\left(f_{l}\right)S\left(f_{l+|1-j|}\right)}{(c-1)LP_{S}}\leq \nu_{2}\\ b\sum_{l=1}^{L}|S\left(f_{l}\right){|}^{2}=P_{s}\\ 0\leq |S\left(f_{l}\right){|}^{2}\leq \frac{P_{s}}{b},l=1,\cdots ,L \end{array},$$

where $f_{l},l=1,\cdots ,L$ are the uniform partition points in the frequency interval W and $b=f_{l+1}-f_{l}$.

When γ=0, $\nu_{1}=1$, and $\nu_{2}=1$, that is, the LPI waveform is optimized without regard to radar detection and resolution performance, the optimized waveform—considering only the interception performance of the PIS—is $|S^{⋆}\left(f\right){|}^{2}=P_{s}/W$, under the assumption that the background noise $n_{2}$ is a white Gaussian noise, whose PSD $P_{N_{2}}\left(f\right)$ is a constant. Thus, in this case we can draw a conclusion that for PISs, it is most difficult to intercept a radar waveform whose power is distributed equally within the whole bandwidth W. When the radar resolution and interception performance of the PIS are not considered, and only the radar detection performance is considered, the radar detection performance metric $D\left(x;n_{1}\right)$ is maximized, and the optimized waveform concentrates all waveform energies at the frequency where $\sigma_{H}^{2}\left(f_{l}\right)/P_{N_{1}}\left(f_{l}\right)$ achieves the maximum value. The optimized LPI waveform in Equation (12) should be a compromise between one optimized solution, i.e., energy is distributed equally in all frequencies, considering only the interception performance of the PIS, and another optimized solution, i.e., energy is concentrated at a certain frequency, considering only radar detection performance.

An efficient method for solving nonlinear constrained optimization problems is the combination of the interior point and sequential quadratic programming (SQP) methods. Here, we use the algorithm proposed by Byrd [18] to solve the optimization problem in Equation (12), which jointly utilizes trust regions to ensure the robustness of iterations.

## 3. Simulation Results

**Figure 1:**
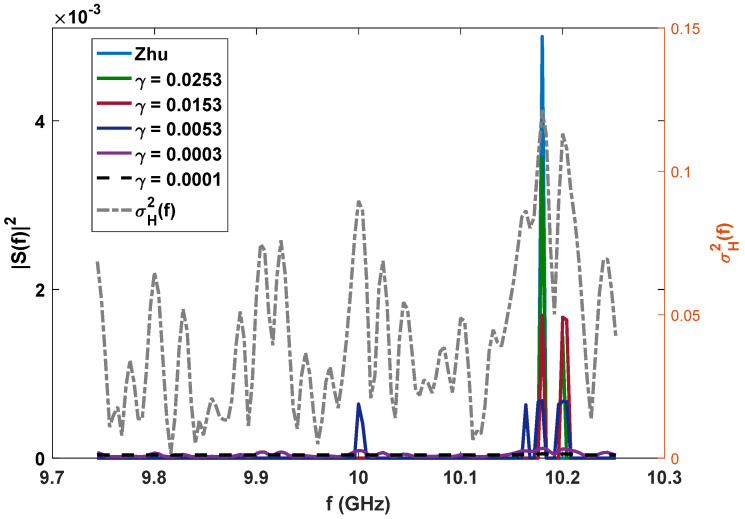
Optimized LPI radar waveforms under the constraint of radar detection performance (white Gaussian noise, $P_{N1}=P_{N2}=1.9531\times 10^{-18}$, $P_{s}=20\;kw$), the variance of target impulse response $\sigma_{H}^{2}\left(f\right)$.

In this section, several simulation experiments are provided. The simulation parameters are: $G_{t}=G_{r}=30\;dB$, $G_{i}=0\;dB$, λ=0.03 m, W=512 MHz, T=25 ns, $L_{1}=-20\;dB$, $L_{2}=-10\;dB$, and R=100 km. We suppose the target heading for the radar is an F-16 aircraft, whose variance $\sigma_{H}^{2}\left(f\right),f\in \left[9.744,10.252\right]\;GHz$ with azimuth $0.05^{{}^{\circ}}$ and elevation $5^{{}^{\circ}}$ between the radar and the target has been calculated by electromagnetic software, which is shown as the grey dotted line in Figure 1.

In order to verify the superiority of our proposed LPI radar waveform design method, SNR loss is treated as a performance degradation metric for radar and the PIS, which can be defined as $\delta_{snr}=snr_{opt}-snr_{pro}$, where $snr_{opt}$ is the output SNR for the optimized waveforms proposed by Zhu et al. [12], which just considers the maximization of radar detection performance, and $snr_{pro}$ is the output SNR for the proposed optimized LPI radar waveforms, which not only consider optimal radar detection performance, but also consider the resolution performance of the radar and interception performance of the PIS. The output SNRs are obtained by matched filtering of the radar and time–frequency analysis of the PIS.

### 3.1. LPI Waveform Design Considering Radar Detection Performance and the PIS

**Figure 2:**
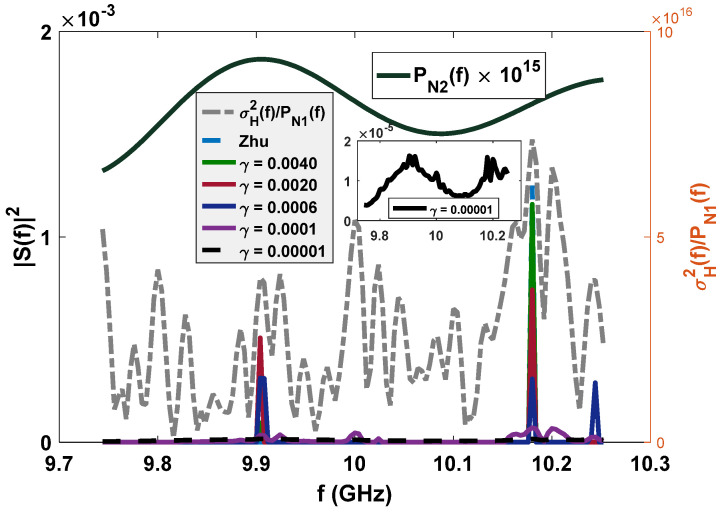
Optimized LPI radar waveforms under the constraint of the radar detection performance (colored Gaussian noise, $P_{N1}=P_{N2}$, $P_{s}=5\;kw$), target-to-noise ratio $\sigma_{H}^{2}\left(f\right)/P_{N1}\left(f\right)$ of radar, PSD of colored Gaussian noise $P_{N2}\left(f\right)$.

The baby-blue dotted lines (only one-sided PSDs are shown) in Figure 1 (experiment 1: white Gaussian noise) and Figure 2 (experiment 2: colored Gaussian noise) are the optimal radar waveforms for target detection proposed by Zhu et al. [12], which place all their power at the frequency where $\sigma_{H}^{2}\left(f\right)/P_{N1}\left(f\right)$ (denoted by $C\left(f\right)$) is maximum. The aim of the proposed LPI waveform design method is to reduce the peak power under a certain loss of radar detection and resolution performance and a fixed transmission power, in order to decrease the interception performance of the PIS. That is, some power will be placed at other frequencies. In this subsection, we first consider radar detection performance, and the combination of radar detection and resolution performance is considered in the next subsection.

***For experiment 1***, since $P_{N2}\left(f\right)$ is a constant, only by putting some power at the frequency where C(f) is the secondary maximum can we furthest reduce the peak power to minimize $D\left(z;n_{2}\right)$ under a given radar detection performance constraint γ, as the green and red lines show in Figure 1. With the relaxing of constraint γ, the power of the optimized LPI waveform will be put at the corresponding frequencies in a descending order of $C\left(f\right)$ (as the deep blue and purple lines show in Figure 1) until the energy is distributed equally within the whole bandwidth (as the black dotted line shows in Figure 1). ***For experiment 2***, since $P_{N2}\left(f\right)$ is not a constant anymore, the frequencies at which the power reduced from the baby-blue dotted line can be placed are related to both $C\left(f\right)$ and $P_{N2}\left(f\right)$. As the light green and red lines show in Figure 2, the reduced power is first placed at the frequency where $P_{N2}\left(f\right)$ is maximum, which can minimize the $D\left(z;n_{2}\right)$ in Equation (10). With the decrease in the value of the radar detection performance constraint γ, the optimized LPI waveforms are the result of the combined effects of $P_{N2}\left(f\right)$ and $C\left(f\right)$ (as the deep blue and purple lines show in Figure 2). When γ tends to zero, the optimized waveform is almost completely influenced by $P_{N2}\left(f\right)$, and they have the same shape, as the black dotted line shows in Figure 2.

**Figure 3:**
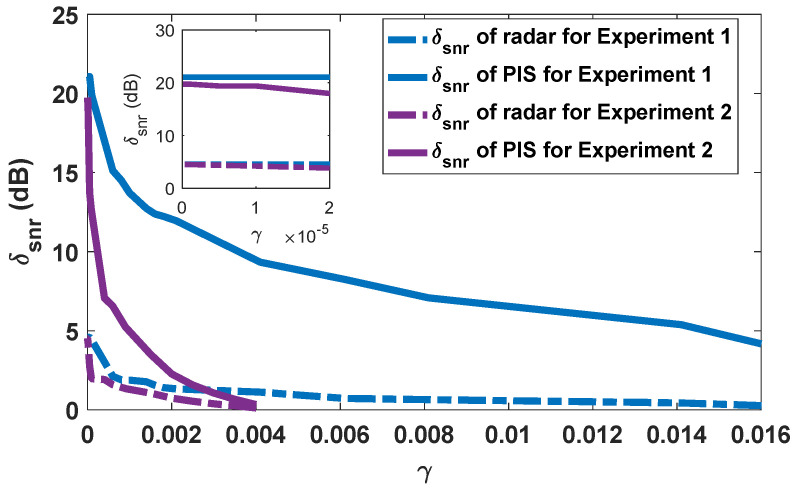
Performance degradation versus radar detection performance constraint.

Since the PIS has no prior information about the transmitted waveform, a reduction in peak power may have a serious effect on its output SNR. In contrast, radar can reduce the effect significantly using the matched filtering technique. As shown in Figure 3, with the decrease in radar detection performance constraint γ, the performance degradation $\delta_{snr}$ gradually increases and remains unchanged in the end. The degradation of radar performance for experiment 1 and 2 stays within 5 dB, while that of the PIS can finally reach 20 dB. As there is such a large performance degradation gap between the radar and the PIS, the optimized radar waveform can achieve a superior LPI performance.

### 3.2. LPI Waveform Design Considering Radar Detection and Resolution Performance and PIS Interception Performance

**Figure 4:**
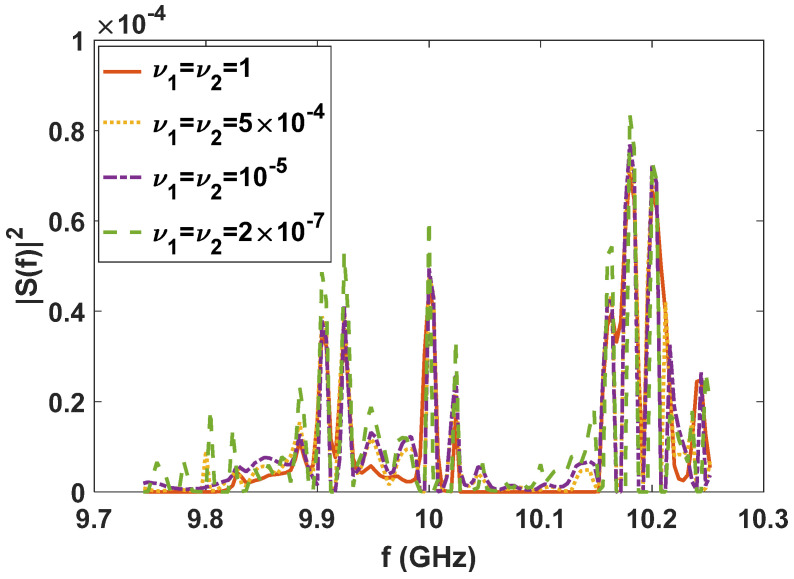
Optimized LPI radar waveforms under the constraint of radar detection and resolution performance (colored Gaussian noise, $P_{N1}=P_{N2}$, $P_{s}=5\;kw$, γ=0.0001; constrained normalized Doppler shifts are in [−2,2]; constrained normalized time delays are in [−0.1575,0.1575]).

**Figure 5:**
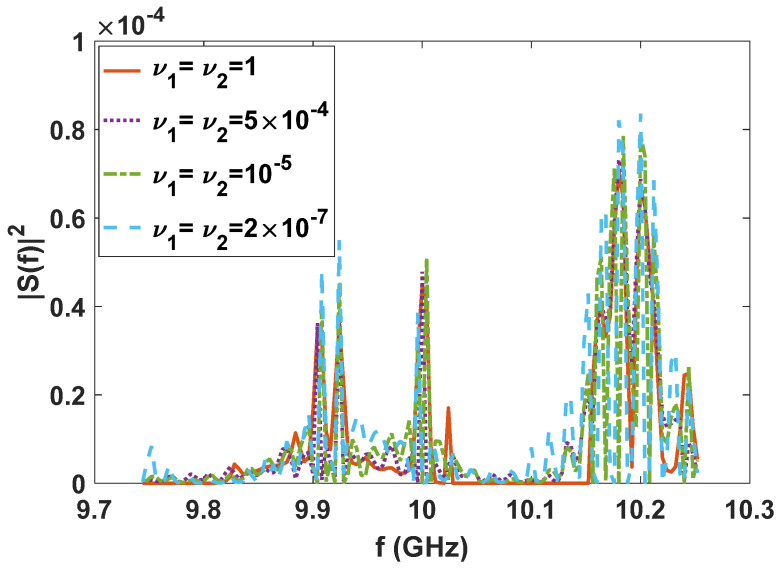
Optimized LPI radar waveforms under the constraint of radar detection and resolution performance (colored Gaussian noise, $P_{N1}=P_{N2}$, $P_{s}=5\;kw$, γ=0.0001; constrained normalized Doppler shifts are in [−3,3]; constrained normalized time delays are in [−0.2362,0.2362]).

Since the LPI waveforms (which are designed to minimize the interception performance of a PIS with a certain loss of radar detection performance) do not have good range and velocity resolution, we also need to further design LPI radar waveforms to satisfy a given requirement of radar resolution performance. In this subsection, we further optimize the LPI radar waveforms, which are designed in *experiment 2* of Section 3.1, to meet the requirements of radar detection and resolution performance simultaneously. Under the background of colored Gaussian noise, whose PSD is displayed in Figure 2, the designed LPI waveforms are shown in Figure 4 with different values of resolution constraints $\nu_{1}$ and $\nu_{2}$, and a given radar detection performance constraint γ=0.0001. In these designs, the constrained normalized Doppler shifts are in the interval [−2,2], and the constrained normalized time delays are in the interval [−0.1575,0.1575], the length of which can be set according to the actual demands, which is the reflection of parameter c in Equation (8). As shown in Figure 4, the power of these optimized LPI waveforms has been put at the frequencies where $C\left(f\right)$ has a local extremum to implement the maximization of the radar detection performance. In Figure 4, we can also find that the smaller the value of the resolution performance constraints $\nu_{1}$ and $\nu_{2}$, that is, the higher the requirement level of radar resolution performance, the more the power is put at the frequencies where $C\left(f\right)$ has a local extremum. This is because it is meant to satisfy the higher requirement of resolution performance by sacrificing more LPI performance, that is to put more power at the frequencies where $C\left(f\right)$ has a local extremum, under a certain requirement γ=0.0001 of radar detection performance. There are the same conclusions when the constrained normalized Doppler shifts and normalized time delays are extended to the intervals [−3,3] and [−0.2362,0.2362], respectively, as Figure 5 shows.

**Figure 6:**
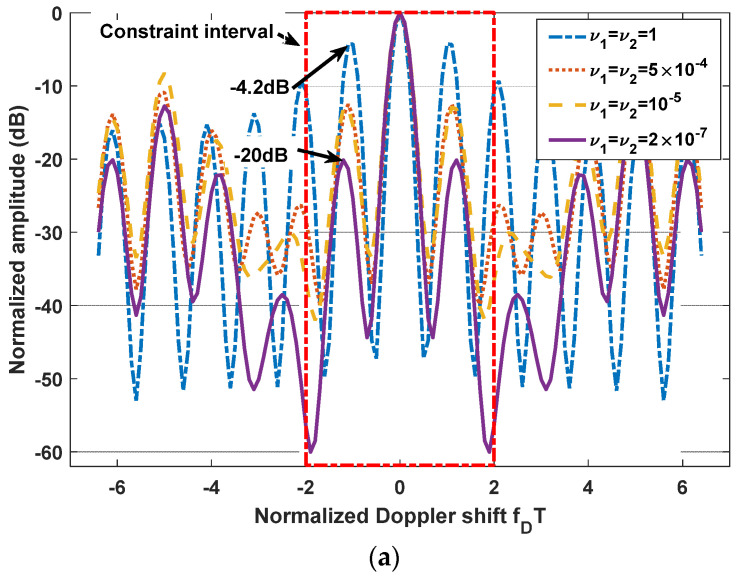

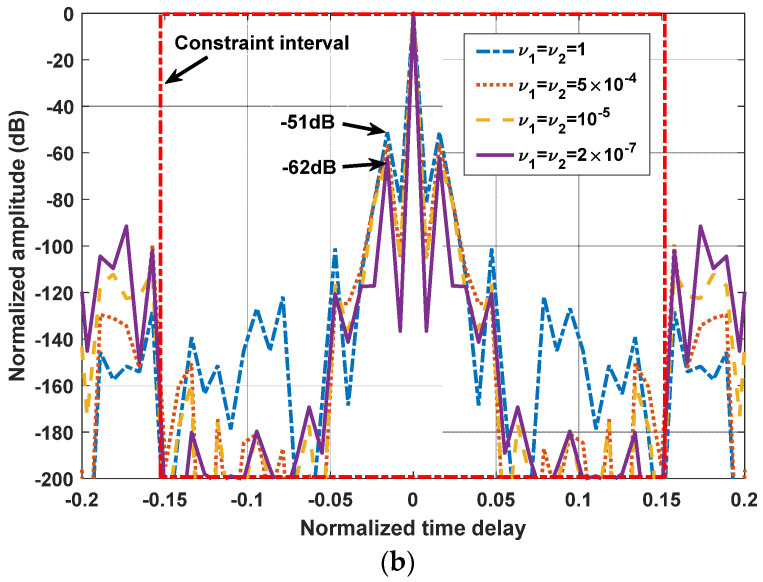
(**a**) One-dimensional zero-delay cuts of the ambiguity function of optimized LPI radar waveforms; (**b**) One-dimensional zero-Doppler cuts of the ambiguity function of optimized LPI radar waveforms; (colored Gaussian noise, $P_{N1}=P_{N2}$, $P_{s}=5\;kw$, γ=0.0001; constrained normalized Doppler shifts are in [−2,2]; constrained normalized time delays are in [−0.1575,0.1575]).

**Figure 7:**
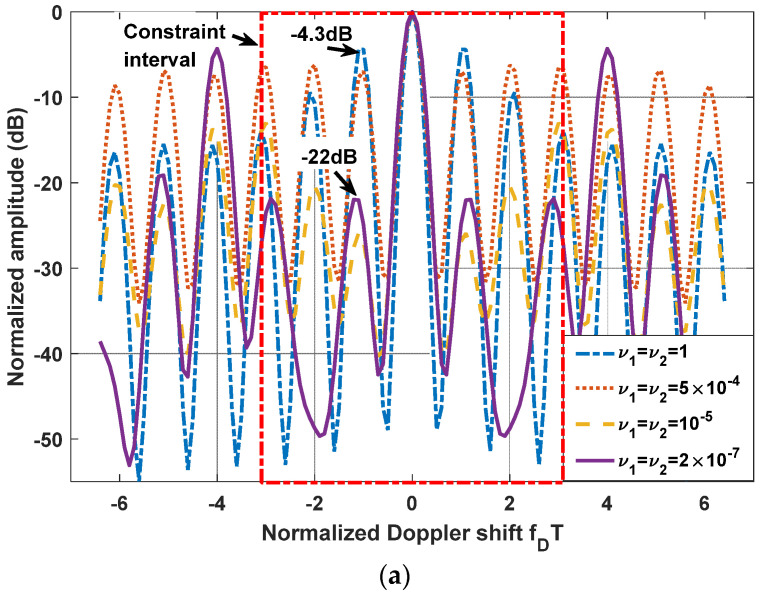

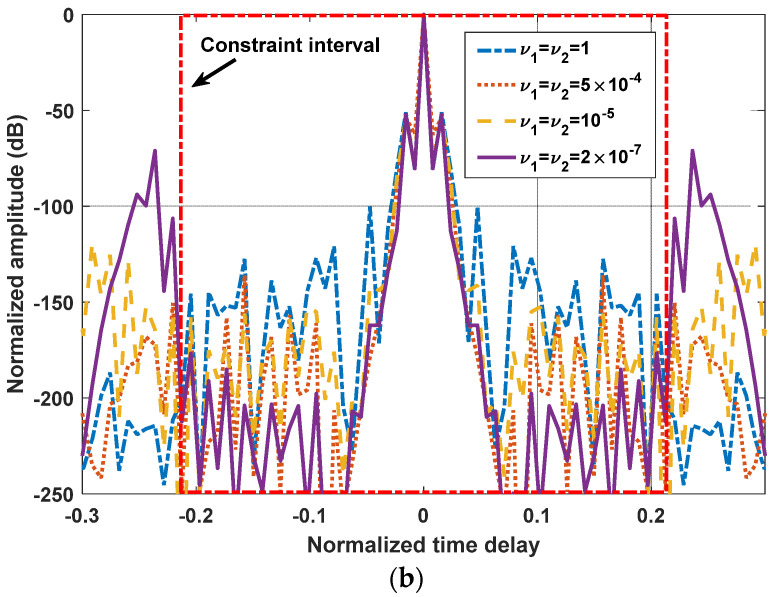
(**a**) One-dimensional zero-delay cuts of the ambiguity function of optimized LPI radar waveforms; (**b**) One-dimensional zero-Doppler cuts of the ambiguity function of optimized LPI radar waveforms; (colored Gaussian noise, $P_{N1}=P_{N2}$, $P_{s}=5\;kw$, γ=0.0001; constrained normalized Doppler shifts are in [−3,3]; constrained normalized time delays are in [−0.2362,0.2362]).

**Figure 8:**
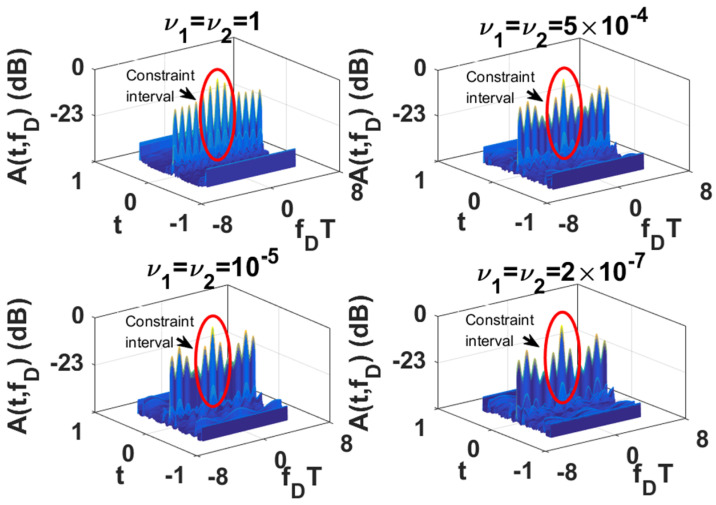
Ambiguity function of optimized LPI radar waveforms for different resolution performance constraints $\nu_{1}$ and $\nu_{2}$ (colored Gaussian noise, $P_{N1}=P_{N2}$, $P_{s}=5\;kw$, γ=0.0001; constrained normalized Doppler shifts are in [−2,2]; constrained normalized time delays are in [−0.1575,0.1575]).

**Figure 9:**
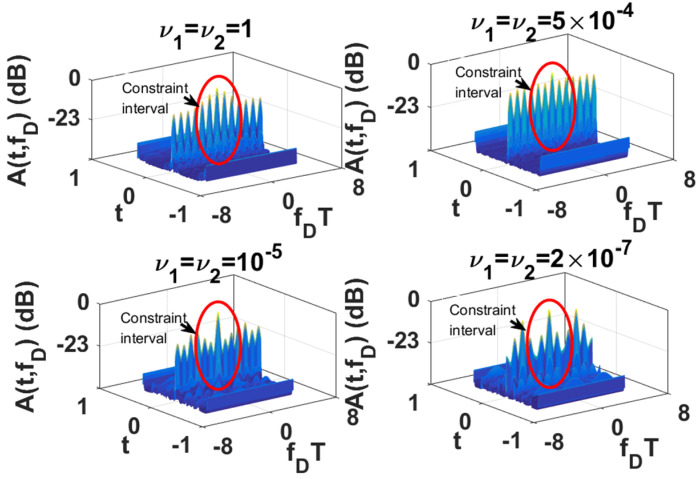
Ambiguity function of optimized LPI radar waveforms for different resolution performance constraints $\nu_{1}$ and $\nu_{2}$ (colored Gaussian noise, $P_{N1}=P_{N2}$, $P_{s}=5\;kw$, γ=0.0001; constrained normalized Doppler shifts are in [−3,3]; constrained normalized time delays are in [−0.2362,0.2362]).

**Figure 10:**
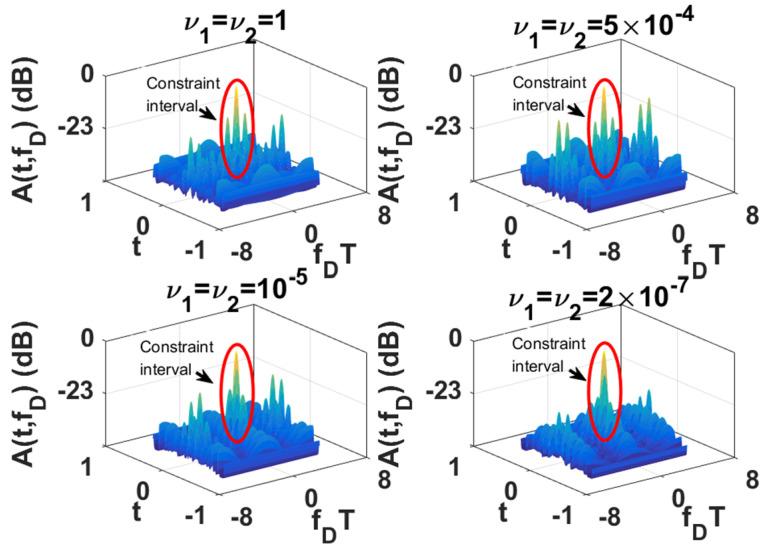
Ambiguity function of optimized LPI radar waveforms for different resolution performance constraints $\nu_{1}$ and $\nu_{2}$ (colored Gaussian noise, $P_{N1}=P_{N2}$, $P_{s}=5\;kw$, γ=0.0005; constrained normalized Doppler shifts are in [−2,2]; constrained normalized time delays are in [−0.1575,0.1575]).

The one-dimensional zero-delay and zero-Doppler cuts of the ambiguity function of these optimized LPI radar waveforms are shown in Figure 6 and Figure 7 for different intervals of constrained normalized Doppler shifts and normalized time delays. Figure 6a displays the one-dimensional zero-delay cuts of the ambiguity function of those optimized LPI radar waveforms, whose constrained normalized Doppler shifts are in the interval [−2,2]. From Figure 6a, we find that the peak values of each sidelobe become smaller and smaller with the fall in the value of resolution performance metrics $\nu_{1}$ and $\nu_{2}$ in the constrained interval [−2,2], which means velocity resolution can be improved effectively under the constraint of resolution performance. From Figure 6a, we can find that the first sidelobe can be suppressed to −20 dB below the maximum of the mainlobe with the resolution performance constraint $\nu_{1}=\nu_{2}=2\times 10^{-7}$, while the first sidelobe is −4.2 dB without a resolution performance constraint. Figure 7a shows the one-dimensional zero-delay cuts of the ambiguity function, whose constrained normalized Doppler shifts are in the interval [−3,3]. In the same way, we can find the sidelobes have better suppression, with reduction of the values of resolution performance metrics $\nu_{1}$, and $\nu_{2}$, in a wider range [−3,3] of normalized Doppler shift. From Figure 7a, we can see that the first sidelobe can be suppressed to −22 dB below the maximum of the mainlobe with the resolution performance constraint $\nu_{1}=\nu_{2}=2\times 10^{-7}$, while the first sidelobe is −4.3 dB without resolution performance constraint. Figure 6b and Figure 7b give the one-dimensional zero-Doppler cuts of the ambiguity function of those optimized LPI radar waveforms with the constrained intervals [−0.1575,0.1575] and [−0.2362,0.2362] of normalized time delay, respectively. In Figure 6b, we see that the sidelobes can acquire better suppression with reduction of the values of the resolution performance metrics $\nu_{1}$ and $\nu_{2}$ in the constrained normalized time delay interval [−0.1575,0.1575], which means the range resolution of radar can be effectively improved under the constraint of resolution performance. From Figure 6b, we see that the first sidelobe can be suppressed from −51 dB to −62 dB below the maximum of the mainlobe with the resolution performance constraint $\nu_{1}=\nu_{2}=2\times 10^{-7}$. In Figure 7b, we have the same result in a wider constrained normalized time delay interval [−0.2362,0.2362]. Thus, we can draw the conclusion that the LPI radar waveforms can be designed to effectively satisfy the given requirements of radar detection and resolution performance, which can also be verified in Figure 8, Figure 9 and Figure 10. These three figures furnish the ambiguity functions with different radar detection performance constraints γ=0.0001 (Figure 8 and Figure 9) and γ=0.0005 (Figure 10), and different constrained intervals for radar resolution performance (Figure 8 and Figure 10: normalized Doppler shifts [−2,2], normalized time delays [−0.1575,0.1575]; Figure 9: normalized Doppler shifts [−3,3], normalized time delays [−0.2362,0.2362]). In each figure, different constraint levels of resolution performance have been simulated, and we can see that the sidelobe in the constraint interval has been effectively suppressed with $\nu_{1}=\nu_{2}=2\times 10^{-7}$ compared to other subfigures, which can be suppressed in more Doppler shifts and time delays by increasing the value of parameter c in Equation (8).

In order to verify the superiority of LPI performance of the designed radar waveforms, we calculate the performance degradations $\delta_{snr}$ for radar and PIS in different constraint parameters and compare the optimized waveforms with a common LPI radar waveform (Frank, P1–P4). In Table 1, we find that there is a huge gap in performance degradation between the radar and the PIS for the optimized waveforms (radar detection constraint γ=0.0001; resolution performance constraint $\nu_{1}=\nu_{2}=2\times 10^{-7}$; optimized waveform 1: constrained normalized Doppler shifts are in [−2,2], constrained normalized time delays are in [−0.1575,0.1575]; optimized waveform 2: constrained normalized Doppler shifts are in [−3,3], constrained normalized time delays are in [−0.2362,0.2362]). The performance degradation of radar is 2.6dB, while the performance degradation of the PIS is 11.2 dB. As there are such huge gaps, the optimized waveforms can achieve a superior LPI performance. Compared with the common LPI radar waveforms, our designed waveforms still have a significant LPI superiority, as shown in Table 1. For each compared LPI radar waveform (Frank, P1–P4), we can find the SNR loss of radar for optimized waveforms is approximately equal to that of the compared waveform, as the second row shows. However, the SNR losses of the PIS are different between compared waveforms and the optimized waveforms. As the third row shows, the SNR loss of the PIS of each compared waveform is nearly 3 dB less than that of the optimized waveforms. Therefore, we can conclude that our designed waveforms can maximize the performance degradations of PISs when they meet the requirements of radar detection and resolution performance.

## 4. Conclusions

In response to the LPI requirements of modern military radar, waveforms with a fixed average power constraint have been designed from the perspective of information flow to minimize the interception performance of a PIS (which is measured by the KLD in this paper) under the condition that both the detection performance and resolution performance of the radar make some concessions. In this paper, we presented a simple information theoretic metric to measure the resolution performance of radar by utilizing the joint entropy theory. Simulations verify the superiority of the designed radar waveforms in radar detection, resolution performance, and the LPI performance.

## Figures and Tables

**Table 1:** SNR losses for the common low probability of intercept (LPI) radar waveforms and optimized waveforms (radar detection constraint γ=0.0001; resolution performance constraint $\nu_{1}=\nu_{2}=2\times 10^{-7}$; optimized waveform 1: constrained normalized Doppler shifts are in [−2,2], constrained normalized time delays are in [−0.1575,0.1575]; optimized waveform 2: constrained normalized Doppler shifts are in [−3,3], constrained normalized time delays are in [−0.2362,0.2362]).

	Optimized Waveform 1	Optimized Waveform 2	Frank	P1	P2	P3	P4
$\delta_{snr}$ of radar (dB)	2.63	2.64	2.63	2.63	2.61	2.63	2.63
$\delta_{snr}$ of PIS (dB)	11.22	11.25	8.15	8.12	7.96	8.12	8.12

## Data Availability

Not applicable.
